# Supplementary material for: Why Do Insects Close Their Spiracles? A Meta-Analytic Evaluation of the Adaptive Hypothesis of Discontinuous Gas Exchange in Insects
Source: Insects. 2022 Jan 22;13(2):117. doi: 10.3390/insects13020117 (PMC8878836; doi:10.3390/insects13020117)
Supplement: Supplementary file 1 [file insects-13-00117-s001.zip › S7 R_Code_Seun_Oladipupo.pdf]

```
install.packages("devtools")
devtools::install_github("MathiasHarrer/dmetar")
library(meta)
library(metafor)
install.packages("devtools")
devtools::install_github("MathiasHarrer/dmetar-master")
devtools::install("C:\\Users\\Oladipupo\\Documents\\")
getwd()
library(dmetar)
install.packages("devtools")
devtools::install_github("MathiasHarrer/dmetar")
R.Version()$version.string
devtools::install("C:\\Users\\Oladipupo\\Documents\\dmetar")
getwd()


###Read File
obj1=read.csv(file.choose())

### Perform Meta-analysis on raw data (mean and SD) (obj. 1 and 2.)
Seun1 <- metacont(Ne,
                    Me,
                    Se,
                    Nc,
                    Mc,
                    Sc,
                    data=obj1,
                    studlab=paste(Author),
                    comb.fixed = FALSE,
                    comb.random = TRUE,
                    method.tau = "SJ",
                    hakn = TRUE,
                    prediction = TRUE,
                    sm = "SMD")
Seun1

###Make a forest plot
forest(Seun1,
       sortvar=TE,
       xlim = c(-1.5,0.5),
       rightlabs = c("g","95% CI","weight"),
       leftlabs = c("Author", "N","Mean","SD","N","Mean","SD"),
       lab.e = "Intervention",
       pooled.totals = FALSE,
       smlab = "",
       text.random = "Overall effect",
       print.tau2 = FALSE,
       col.diamond = "blue",
       col.diamond.lines = "black",
       col.predict = "black",
       print.I2.ci = TRUE,
       digits.sd = 2
)
```

```
### Change Forest Plot to RevMan Layout
forest(Seun1,
       layout = "RevMan5",
       digits.sd = 2)

### Run a subgroup analysis
Order.subgroup<-update.meta(Seun1,
                            byvar=Order,
                            comb.random = TRUE,
                            comb.fixed = FALSE)




### Make a Funnel Plot
funnel(Seun1,xlab = "Hedges' g")
funnel(Seun1,xlab = "g",studlab = TRUE)
funnel(Seun1, xlab="Hedges' g",
       contour = c(.95,.975,.99),
       col.contour=c("darkblue","blue","lightblue"))+
  legend(1.4, 0, c("p < 0.05", "p<0.025", "< 0.01"),bty = "n",
         fill=c("red","blue","lightblue"))


###BIAS ESTIMATION

library(dmetar)
eggers.test(x = Seun1)
trimfill(Seun1)


### create funnel plot with trim-and-fill

Seun1.trimfill<-trimfill(Seun1)
funnel(Seun1.trimfill,xlab = "Hedges' g", contour = c(.95,.975,.99),
         +        col.contour=c("red","blue","lightblue"))+
  + legend(1.4, 0, c("p < 0.05", "p<0.025", "< 0.01"),bty = "n",
           +        fill=c("red","blue","lightblue"))



 ###Read File


####Objective 3: Creating a metric size (slope)

Ighalo =read.csv(file.choose())
var1 = sum(variance*n, data = Ighalo)
var1 <- sum((k-1)*Variance, data = Ighalo)
p.var2 <- sum(n*(lnY - yhats)^2, data = Ighalo)
yhats <- lm(k~x, data = Ighalo)$fitted.values
```

```
p.var1 <- sum((rav*num), data = Ighalo)


p.var1 <- sum((Nc)*var, data = obj1)



setwd("E:/FSH")
write.csv(yhats,"yhats.csv")

###Calculate percent change and variances for loglinear models;

### Input: temperature (x) and natural log of metabolic rate (lnY)
### Output: effect size (% change in metabolic rate per unit change in
temperature),
###        variance of the effect, and sample size
Ighalo =read.csv(file.choose())
ll.reg <- lm(lnY ~ x, data =Ighalo)
ll.sum <- summary (ll.reg)
ll.slope <- ll.sum$coefficients[2,1]
ll.se <- ll.sum$coefficients[2,2]
ll.effect <- 100*(exp(ll.slope) - 1)
ll.var <- 10000*exp (2*ll.slope)*(9.08325E-05^2)
ans <- cbind(ll.effect, ll.var, ll.slope)
colnames(ans) <- paste(c("Effect Size", "VARIANCE", "BETA"))
rownames (ans) <- paste ("")
print (round(ans,6))




###BIAS ESTIMATION

kl <- eggers.test(x = Seun1)
K2 <- cbind(Intercept, ConfidenceInterval, t, p)
colnames(K2) <- paste(c("Intercept", "ConfidenceInterval", "t", 'p'))
print (round(K2,6))

### TO Start a Phylogenetic analysis,

## First clear working environment

rm(list=ls())
### Load Libraries


library(MCMCglmm)
library (ape)
library (phytools)

### Set working directory

setwd("E:/Meta-analysis")
```

```
## Read data File
Ighalo =read.csv(file.choose())

###Create and attach tree
tree <-'(((Phasmatodea,Orthoptera,(Isoptera,(Mantophasmatodea,Blattodea)))),
(Hemiptera,(Coleoptera,(Diptera,Hymenoptera)))) ;'
tree <- read.newick(text=tree)
attach (tree)


### Make tree ultrametric and plot it

tree <- compute.brlen(tree, method="Grafen")
is.ultrametric(tree)
plot(tree)

### Match tips to data

check.species<-function(x) {any(x==tree$tip.label)}
print(check.species)
Ighalo <- Ighalo[sapply(Ighalo[,"Order"],check.species),]
Ighalo <- Ighalo[complete.cases(Ighalo),]
print(head(Ighalo))

### Invert phylogenetic covariance matrix
INtree <- inverseA(tree, nodes="ALL",scale=TRUE, reduced=FALSE, tol
=.Machine$doubleeps^0.5)

### Set priors
prior <- list(R=list(V=1, nu=0.002),
              G=list(G1=list(V=1, nu=1),
                     G2=list(V=1, nu=1), G3=list(V=1, nu=1), G4=list(V=1,
nu=1)))
prior$G

### Run model

model.Order <- MCMCglmm(Change ~ 1,
                        random= ~ Order + Author + Family + Species,
                        data=Ighalo,
                        mev=Ighalo$Variance,
                        prior=prior,
                        nitt=1000000,
                        thin=100,
                        burnin=500000)
summary(model.Order)


### Conduct meta-analysis for objective 3 (see data set for example)


Kemi <- metagen(TE=Change,
                  seTE=Variance,
                  data = Ighalo,
```

```
                  studlab = paste(Author),
                  comb.fixed = FALSE,
                  comb.random = TRUE,
                  method.tau = "SJ",
                  hakn = TRUE,
                  prediction = TRUE,
                  sm = "SMD")

### Forestplot for objective 3
forest(Kemi,
       sortvar=Order,
       xlim = c(-1.5,0.5),
       rightlabs = c("g","95% CI","weight"),
       leftlabs = c("Author", "N","Mean","SD","N","Mean","SD"),
       lab.e = "Intervention",
       pooled.totals = FALSE,
       smlab = "",
       text.random = "Overall effect",
       print.tau2 = FALSE,
       col.diamond = "blue",
       col.diamond.lines = "black",
       col.predict = "black",
       print.I2.ci = TRUE,
       digits.sd = 2)


##Change to RevMan Layout

forest(Kemi,
       layout = "RevMan5",
       digits.sd = 1)

###Run sub-group analysis


order.subgroup<-update.meta(Kemi,
                            byvar=Order,
                            comb.random = TRUE,
                            comb.fixed = FALSE)
order.subgroup
forest(order.subgroup,
       layout = "RevMan5",
       digits.sd = 6)

####Plot funnel

funnel(order.subgroup, xlab="Hedges' g",
       contour = c(.95),
       col.contour=c("lightblue"))+
  legend(1.4, 0, c("p < 0.05"),
         fill=c("lightblue"))

###BIAS ESTIMATION
```

```
library(dmetar)
eggers.test(x = Seun1)
trimfill(Seun1)


### create funnel plot with trim-and-fill

Seun1.trimfill<-trimfill(Seun1)
funnel(Seun1.trimfill,xlab = "Hedges' g", contour = c(.95,.975,.99),
       +        col.contour=c("red","blue","lightblue"))+
  + legend(1.4, 0, c("p < 0.05", "p<0.025", "< 0.01"),bty = "n",
           +        fill=c("red","blue","lightblue"))
```
