# Supplementary figures and images for: Why Do Insects Close Their Spiracles? A Meta-Analytic Evaluation of the Adaptive Hypothesis of Discontinuous Gas Exchange in Insects
Source: Insects. 2022 Jan 22;13(2):117. doi: 10.3390/insects13020117 (PMC8878836; doi:10.3390/insects13020117)

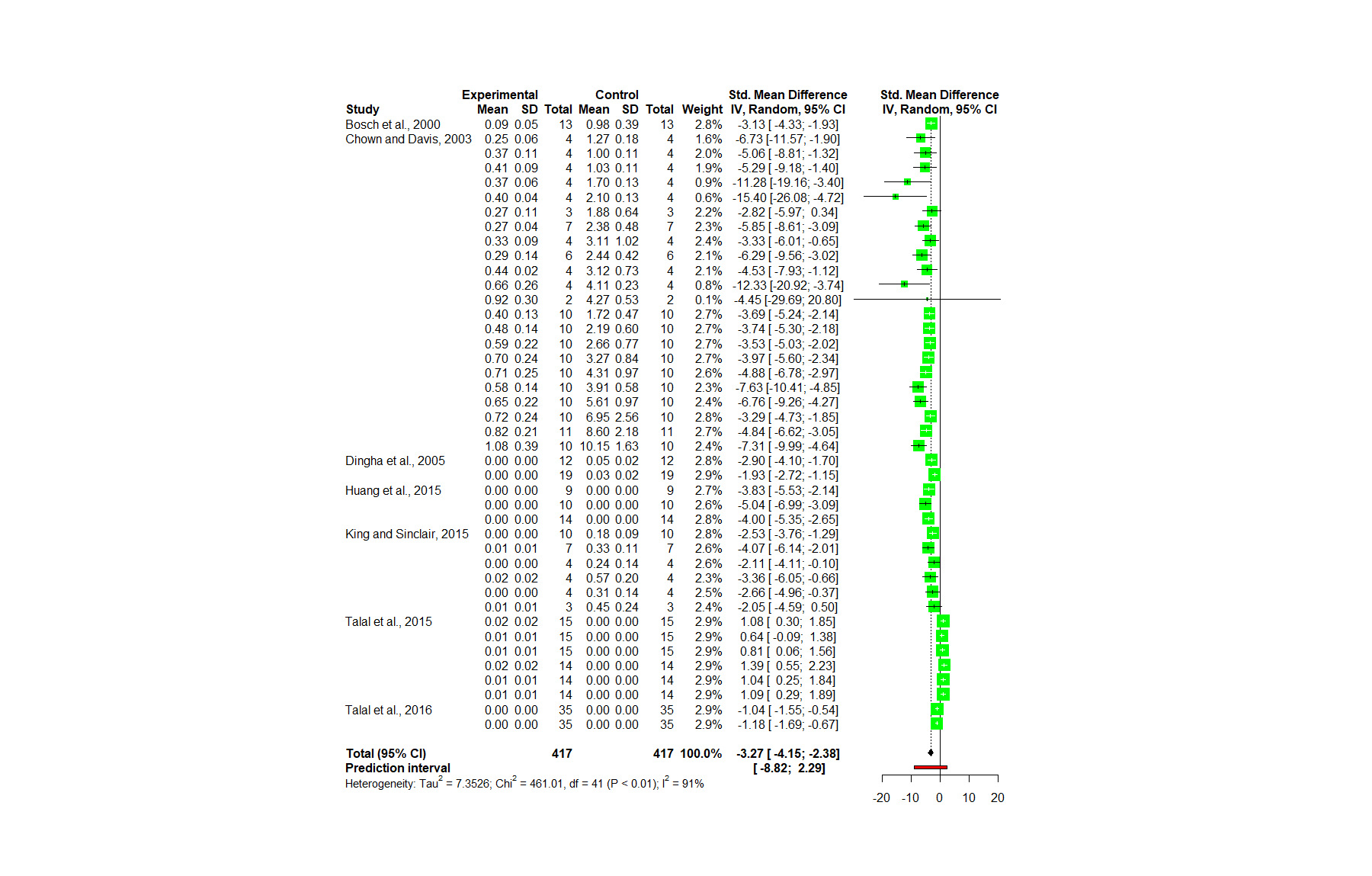

Supplement: Supplementary file 1 [file insects-13-00117-s001.zip › S2 Overall forest Plot for water.png]

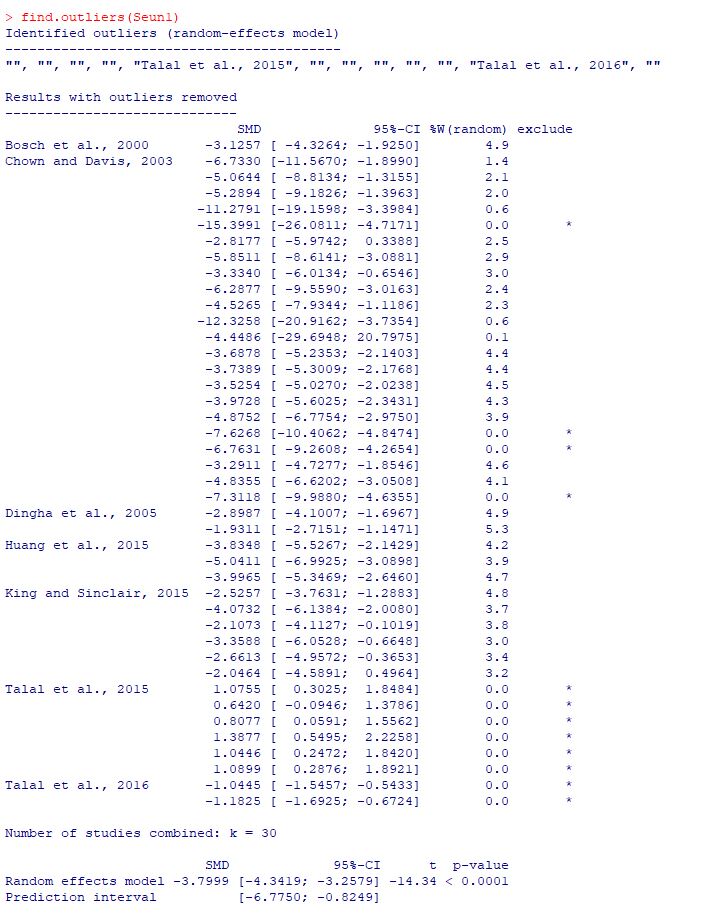

Supplement: Supplementary file 1 [file insects-13-00117-s001.zip › S3a Wateroutlier1.JPG]

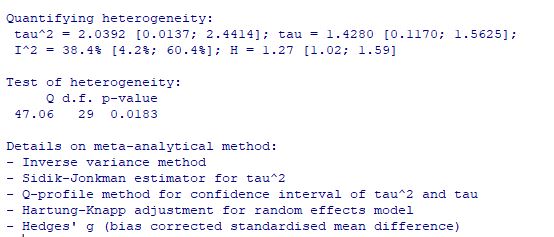

Supplement: Supplementary file 1 [file insects-13-00117-s001.zip › S3b Wateroutlier2.JPG]

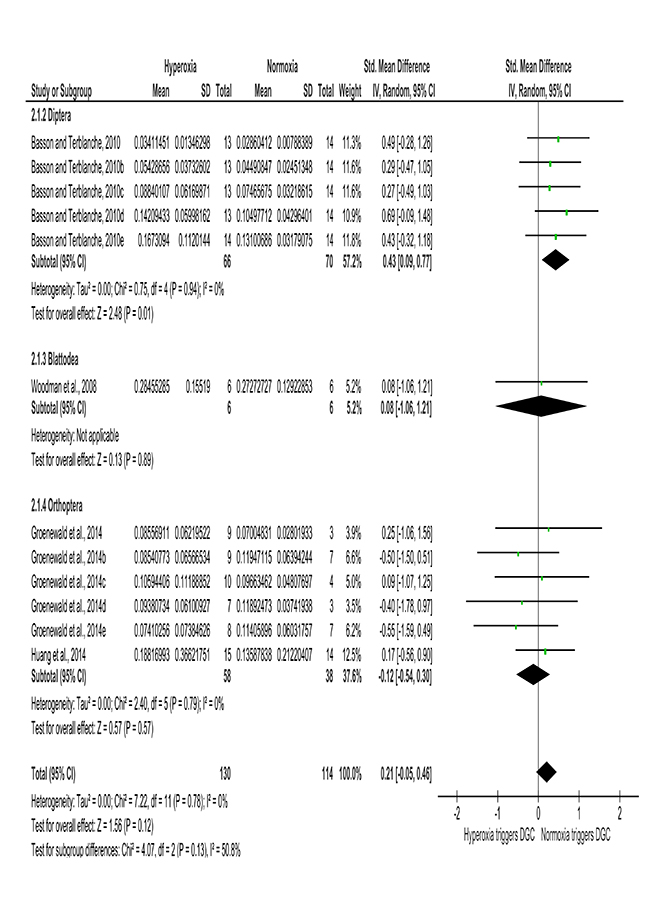

Supplement: Supplementary file 1 [file insects-13-00117-s001.zip › S4 subgroup hyper.jpg]

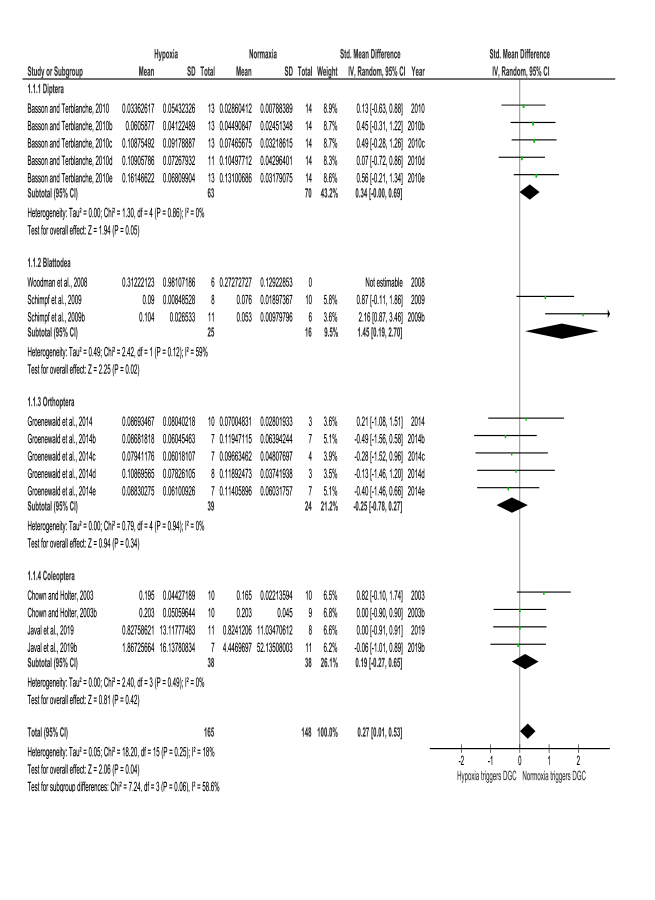

Supplement: Supplementary file 1 [file insects-13-00117-s001.zip › S5 subgroup hypo.jpg]

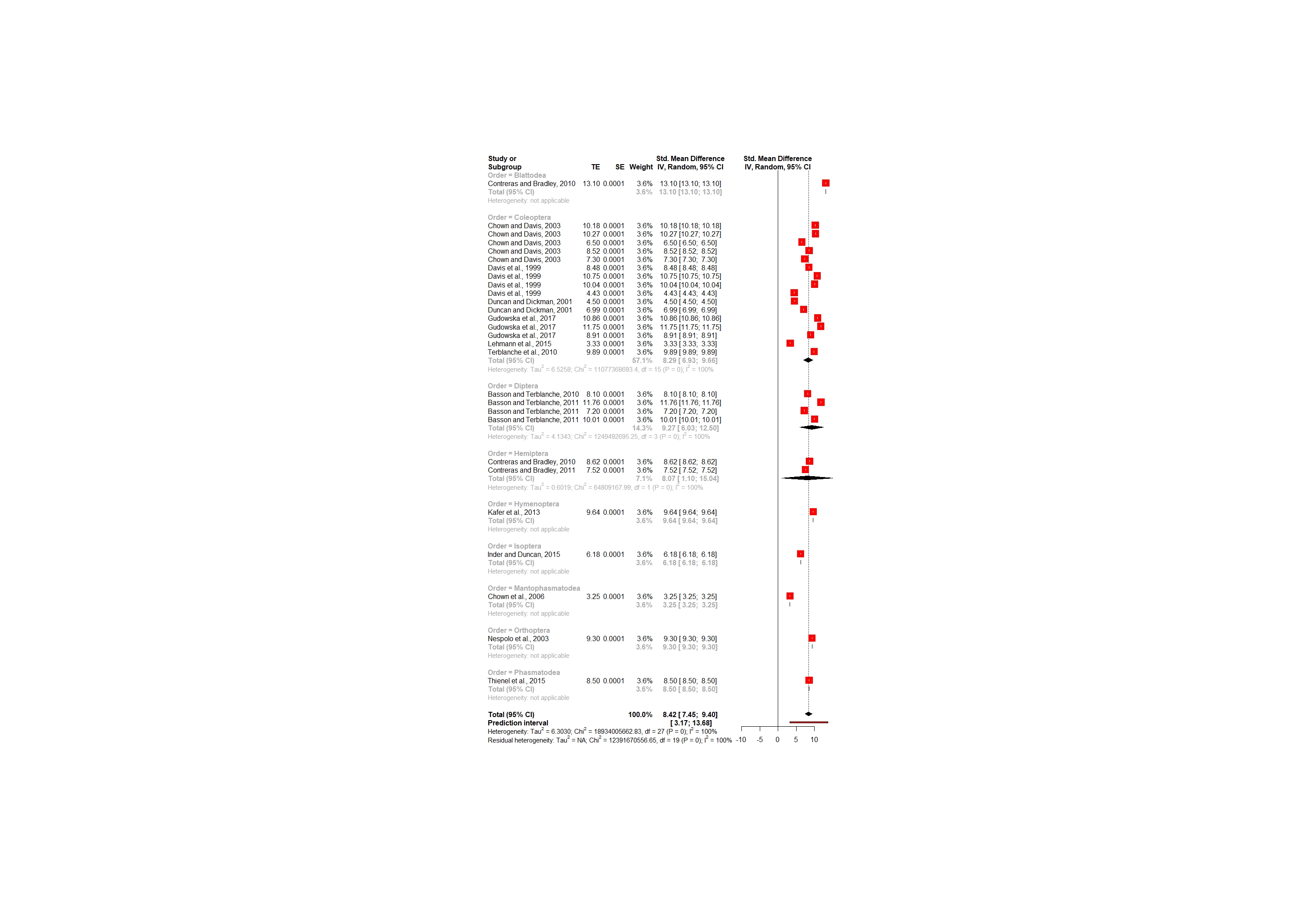

Supplement: Supplementary file 1 [file insects-13-00117-s001.zip › S6 Overall Forest Plot for Temperature.jpeg]
